# Smoothened Inhibitor, PF-04449913 Inhibits the Development of Myelofibrosis in a JAK2V617F Transgenic Mouse Model by Reducing TGF-β and MAPK Signaling Pathways

**DOI:** 10.21203/rs.3.rs-6580439/v1

**Published:** 2025-05-09

**Authors:** Akil Merchant, Parvesh Chaudhry, Mohan Singh, Tucker Lemos, Eman Fargal, Imran Siddiqi, Casey O’Connell, Zhizhuang Zhao

**Affiliations:** Cedars-Sinai Medical Center; University of Southern California; University of Southern California; Cedars-Sinai Medical Center; Tanta University; University of Southern California–Keck School of Medicine; University of Southern California; Uni of Oklahoma Health Sciences

## Abstract

Treatment of JAK2V617F driven myeloproliferative neoplasms (MPNs) with Ruxolitinib (JAK inhibitor, JAKi) has shown limited disease-modifying benefits and has led to the search for other pathways as potential therapeutic targets for this disease. We investigated the effects of Smoothened inhibition (SMOi) using the small-molecule inhibitor PF-04449913 (PF-13) in a JAK2V617F transgenic mouse model that recapitulates many of the phenotypes of MPNs including bone marrow fibrosis and splenomegaly. We show both that hedgehog (Hh) signaling pathway is activated in JAK2V617F cells and that SMOi reduces splenomegaly in JAK2V617F mice. In a murine bone marrow transplant model, we show that SMOi also reduces JAK2V617F allelic burden. JAK2V617F mice show increased pERK and NF-κB signaling, which is reduced with SMOi. Finally, we found that SMO inhibitor blocks bone marrow fibrosis by reducing TGF-β signaling. In conclusion, this report provides critical insight into the mechanism of action of SMO inhibitors in JAK2V617F associated MPN.

## INTRODUCTION

The Janus Kinase (JAK) - Signal Transducer and Activator of Transcription (STAT) pathway plays a critical role in transmitting signals from several cytokines and growth factors into the nucleus to regulate gene expression[1]. The JAK2/STAT signaling pathway is activated upon binding of a cytokine to its receptor, causing phosphorylation and activation of JAK2, a nonreceptor tyrosine kinase, which then recruits and phosphorylates STATs, which dimerize, translocate to the nucleus, and activate target gene transcription. Genetic alterations in JAK/STAT pathway are associated with several pathologies including hematological malignancies[2]. The JAK2 gain-of-function mutation, JAK2V617F, was first described in 2005 and is highly prevalent in myeloproliferative neoplasms (MPNs)[3–6]. This mutation is seen in 95–100% of patients with polycythemia vera (PV) and in about 50% of patients with essential thrombocytosis (ET) and primary myelofibrosis (MF)[6, 7]. MF can occur *de novo* or secondary to other related MPNs, including PV (post-PV MF) or ET (post-ET MF)[5, 8]. JAK2V617F mutation results in constitutive activation of the downstream signaling pathways such as STAT3/STAT5, RAS/MEK/ERK, and PI3K/AKT[5, 9, 10]. Currently, the only potentially curative therapy for patients with MF is allogeneic hematopoietic stem cell transplantation (HSCT)[11]. Due to treatment-related morbidity and mortality, HSCT is only recommended for patients with intermediate-2– or high-risk disease who are fit enough to undergo the procedure. The majority of patients with MF are treated with palliative therapies, which improve disease symptoms rather than altering the natural history of disease[12].

Approval of Ruxolitinib, a selective JAK1/JAK2 inhibitor by the US FDA in 2011 changed the therapeutic landscape of MF. Clinical approval of Ruxolitinib was based on the results from phase 3 randomized COMPORT-I and -II trials where Ruxolitinib provided significant and sustained reduction in splenomegaly over the best available therapy (BAT) or placebo group[13, 14]. However, soon it was recognized that Ruxolitinib is not a curative therapy and has a limited disease modifying effect because Ruxolitinib does not improve bone marrow fibrosis and provide limited reduction of JAK2V617F allelic burden[12]. Ruxolitinib appears to block inflammatory cytokine activity rather than stem cell–derived clonal myeloproliferation, which is the primary driver of the disease[15]. Therefore, disease resistance can ensue following an initial response to JAK2 inhibition[15]. In addition, treatment-related anemia may exacerbate preexisting MF-related anemia[13, 14, 16]. This limited effect is related to the fact that Ruxolitinib, like the other type I JAK2 inhibitors, equally inhibits wild-type JAK2 (JAK2 WT) and aberrant JAK2 associated with oncogenic activation[17]. These data clearly suggest that JAK inhibitor monotherapy incompletely addresses the burden of JAK2V617F associated disease. The recently approved agents, Pacritinib and Momelotinib, are also JAK inhibitors with similar mechanisms of action. Therefore, there is a need to identify other signaling pathways that can be targeted in JAK2V617F patients in combination with JAK2 inhibitors.

The hedgehog (Hh) pathway is an essential signaling pathway in the maintenance or homeostasis of hematopoietic precursors[18–21], and is thus an ideal candidate for targeting in MPN[22]. The Hh pathway is a highly conserved signaling pathway, including cell surface receptor Patched-1 (PTCH1), transmembrane receptor Smoothened (SMO), and three glioma associated family transcription factors (GLI1, GLI2, and GLI3)[23, 24]. In the absence of a Hh ligand, PTCH1 inhibits SMO, and GLI transcription factors are sequestered into the cytoplasm and processed into a repressor form (GLI2R and GLI3R), which translocates to the nucleus to inhibit transcription of Hh pathway target genes. In the presence of a Hh ligand, PTCH-mediated inhibition of SMO is relieved, and SMO activates the signaling cascade. Activated GLIs then translocate to the nucleus to regulate target gene transcription. Therefore, the output of Hh signaling is controlled by the ratio of activator and repressor GLI proteins[25]. To date, preclinical data on the potential role of the Hh pathway in MF are limited. There are several lines of evidence that suggest a rationale for targeting the Hh pathway in MF. For example, the expression of *Gli1* and *Ptch1* was increased up to 100-fold in granulocytes isolated from patients with MPNs compared with control granulocytes[26] Further in this study it was shown that SMO inhibitor Sonidegib (LDE-225) in combination with Ruxolitinib significantly reduced blood counts, mutant allele burden, and bone marrow fibrosis as compared to that observed with Ruxolitinib as monotherapy[26]. Among 23 intermediate/high-risk MF patients treated with the combination of these same two agents in the phase 1b portion of a phase 1b/2 study, 65% achieved a ≥ 50% reduction in palpable spleen length from baseline[27]. In a phase 1a study of SMO inhibitor PF-04449913 (PF-13) for patients with hematological malignancies (n = 32), five of the six MF patients attained stable disease, and the sixth stayed on study for over a year while achieving durable clinical improvement with a > 50% reduction in extramedullary disease[28]. IPI-926 was tested in a phase 2 study as a single agent in patients with myelofibrosis[29]. Although it did not meet its primary endpoint, reductions in splenomegaly were observed in 12 of the 14 patients, and one patient achieved transfusion independence for five months.

A growing body of evidence suggests that inhibition of Hh pathway can slow or halt fibrosis, observations further supported by the fact that Smoothened inhibition (SMOi) shows clinical benefit in several fibrotic diseases such as biliary, pancreatic, kidney and lung fibrosis[30–34]. Moreover, activated Hh signaling is also involved in liver fibrosis[35]. In a pre-clinical study, SMOi via the small molecule Vismodegib significantly decreased liver myofibroblasts and progenitors, reduced liver fibrosis, and attenuates early hepatic fibrosis[36, 37]. *In vivo* studies by Jung et al. (2011), provide evidence that Hh ligands induce pancreatic fibrosis via TGF-β and matrix metalloproteinases[33]. Inhibition of Hh pathway by targeting either SMO by LDE223 or GLI1 by GANT61, decreased pulmonary fibrosis and collagen accumulation in a lung injury model[38, 39]. A clinical study combining the Hh pathway inhibitor Sonidegib with the JAK2 inhibitor Ruxolitinib in patients with MF is currently underway (NCT01787552). Based on these earlier findings, the present study was designed to assess the therapeutic effects of SMO inhibitor, PF-04449913 in a transgenic mouse model of myeloproliferative disease as well as possible molecular mechanisms underlying the effects, including its effects on splenomegaly, bone marrow fibrosis, allelic burden and alteration in cellular signaling pathways, and more importantly, its effects on the Hh signaling pathway in a JAK2V617F mouse model.

## MATERIALS AND METHODS

### Mice and Treatments

JAK2V617F transgenic mice were crossed with C57BL/6 mice for over 10 generations, and homozygous transgenic mice were used in the study. JAK2V617F transgenic mice were generated by expressing entire coding region of human JAK2V617F plus the 3’ non coding region in the hematopoietic system under the control of the *vav1* promoter[40]. Animals were housed in ventilated cages under standard conditions. This study was carried out under an approved protocol by the University of Southern California Institutional Animal Care and Use Committee. Wild-type (WT) or JAK2V617F mice (8-to-10 weeks old) were treated with vehicle or PF-04449913 (PF-13) [100 mg/kg body weight (BW)] once daily for five days by oral gavage. Following PF-13 treatment, spleens were removed and weighed using a standard analytical balance from Mettler Toledo (Oakland, CA; USA). Single-cell suspensions from the spleen were stained with Gr-1, B220, CD3, and Ter119. Antibodies for flow cytometry were purchased from BD Biosciences (San Jose, CA; USA). For histopathology analyses, femurs were fixed in formaldehyde, decalcified using Decalcifying Solution-Lite from Sigma-Aldrich (St. Louis, MO; USA), and paraffin embedded for reticulin staining. Reticulin staining was performed using the Accustain Reticulin Stain kit from Sigma-Aldrich.

### Western Blotting

Whole cell lysates were prepared using RIPA lysis buffer with added phosphatase inhibitor and protease inhibitor cocktail (Indianapolis, IN; USA), and immnunoblotting for Hh pathway members (SHH, SMO, GLI1, and GLI3) was done as described previously[41]. Antibodies against SHH, SMO, and TGF-β were from Abcam (Waltham, MA; USA). GLI1 antibody was from Boster Bio (Pleasanton, CA; USA), and GLI3 was procured from R&D Systems (Minneapolis, MN; USA). Antibodies for AKT, pAKT, ERK1/2, pERK 1/2, JAK2, and pJAK2, were obtained from Cell Signaling Technologies (Beverly, MA; USA). Equal amounts of proteins were electrophoresed in NuPAGE^™^ 4–12% Bis-Tris Protein Gels, 1.0 mm, 10-well from Thermo Fisher Scientific (Carlsbad, CA; USA). Proteins were transferred to nitrocellulose membranes using Trans-Blot^®^SD semi-dry transfer cell from Bio Rad Laboratories (Hercules, CA; USA). GAPDH from Thermo Fisher Scientific was used as a loading control. Blots were scanned using the LI-COR Odyssey CLx scanner (Lincoln, NE; USA) set at “Auto,” and quantitation was done using LI-COR Image Studio software.

### Real-Time Quantitative Polymerase Chain Reaction (qPCR)

Total RNA was extracted with Qiagen RNeasy Mini kit (Valencia, CA; USA), and 1 μg was reverse-transcribed using Moloney murine leukemia virus (M-MLV) reverse transcriptase from Invitrogen (Carlsbad, CA; USA) with oligo(dT) primer. Real-time polymerase chain reaction (qPCR) was performed with an Applied Biosystems StepOne^™^ instrument using TaqMan probes for *Col1a1* and *Col2a1* from Applied Biosystems (Foster City, CA; USA). For each sample, gene expression was normalized to murine β-actin and compared using the delta-delta Ct method. Samples were considered negative for expression if Ct values were higher than 40.

### Bone Marrow Transplant Studies

For bone marrow transplantation assays, recipients were C57BL/6 congenic strain that carries the differential *Ptprc*^*a*^ pan leukocyte marker commonly known as CD45.1 or Ly5.1. Recipient mice were sub-lethally irradiated (2 × 450 cGy) before transplantation. Donor cells were derived from 8 week old JAK2V617F transgenic mice or WT mice which express the *Ptprc*^*b*^ (CD45.2 or Ly5.2) allele. For competitive repopulation assays, competitor bone marrow (BM) cells were obtained from recipient CD45.1 mice. A total of 1 × 10^6^ nucleated BM cells at a test/competitor ratio of 4:1 were injected into recipient mice. Ten mice received JAK2V617F BM cells (5 female and 5 male) and ten mice received WT BM cells (5 female and 5 male). Blood was obtained 4 weeks after transplantation and was stained for CD45.1, CD45.2, Gr1, B220, and CD3. After confirming engraftment, mice were randomly divided into four groups for vehicle and PF-13 treatment. The four groups were as follows: (1) JAK2 WT BM + vehicle, (2) JAK2 WT BM + PF-13, (3) JAK2V617F BM + vehicle, and (4) JAK2V617F BM + PF-13. Recipient mice were treated with PF-13 (100 mg/kg BW) or vehicle for four weeks. Allelic burden was measured in peripheral blood by staining for CD45.1 and CD45.2. Additionally, we measured Gr1, B220 and CD3 staining in CD45.2 positive cells.

Cytokine levels were measured using the Mouse 32-Plex Cytokine Array (ab133994) from Abcam following the manufacturer’s protocol. Samples were added to the membrane and incubated for 2 h at room temperature. The membrane was then washed, and cytokine values were obtained using densitometry, with specific concentrations being extrapolated from positive and negative controls as provided by the manufacturer.

### Co-culture with OP9 cells

Mouse stromal OP9 cells were plated at 1 × 10^6^ concentrations in a six-well plate. Splenocytes from WT or JAK2V617F mice were cultured with OP9 stroma for 48 h. After 48 h, RNA was isolated and cDNA synthesis was performed as described above. Quantitative real-time PCR was performed for the expression of *Col1a1, Col2a1,* and *β-Actin*. TaqMan probe primers for qPCR were purchased from Applied Biosystems. The TaqMan^®^ Gene Expression Assay from Thermo Fisher Scientific, was used for qPCR to verify differential gene expression on an Applied Biosystems 7300 Real-Time PCR System. Values obtained were normalized to the housekeeping *β-Actin* gene expression and relative expression compared to control samples, and was calculated by the 2ΔΔCT method. All assays were performed in triplicate.

### Imaging Mass Cytometry (IMC) and Patch Analysis

Clinical samples of MPN patients were baked, dewaxed, and rehydrated as described[42]. Samples were stained with a panel of antibodies, including SHH (EP1190Y) and TGF-β (1D11.16.8). We ablated the samples using Standard BioTools Hyperion Imaging Mass Cytometry System (San Fransisco, CA; USA). Image analysis was performed in patches of 50 μm, and patches were scaled to the 99th percentile of brightness by DNA, Shh, and TGF-β individually, with outliers being discarded. Patches with low to no signal were removed, and patches were split into TGF-β high and TGF-β low regions by a local minima in TGF-β density plot at 0.2. Patches were then plotted by grade and TGF-β-high/low versus Shh signal.

### Statistical Analysis

Differences between two groups were statistically analyzed using Student’s unpaired, two-tailed t-tests. For all bar graphs, data are presented as mean standard error of the mean (SEM). A p-value of less than 0.05 was considered significant.

## RESULTS

### Treatment with SMO antagonist reduces splenomegaly

**Figure 1:**
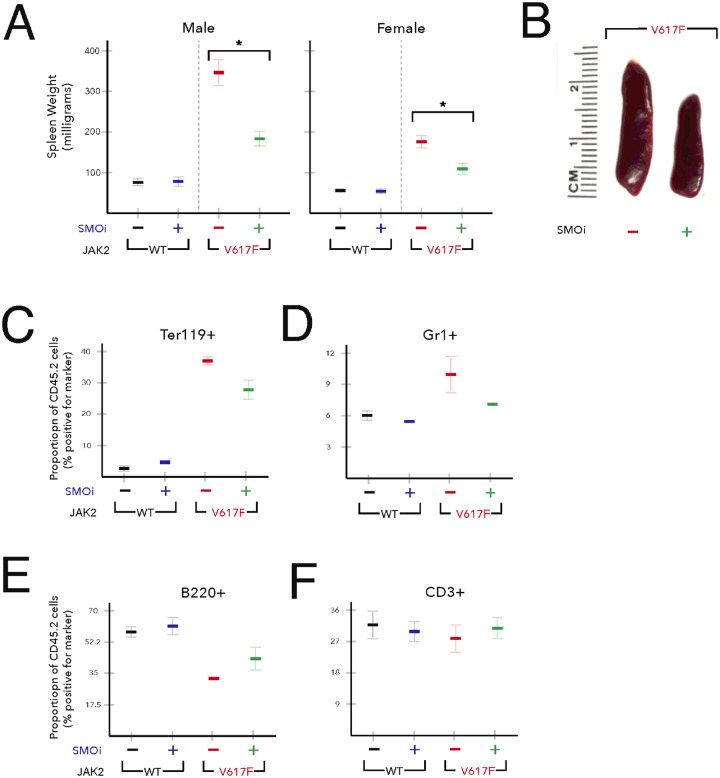
Treatment with SMO inhibitor PF-13 improves splenomegaly. (A-C) 8 week old WT or JAK2V617F mice were treated with vehicle or PF-13 (100 mg/kg) for five days (n=5 each group). Avg. mass +/− SEM*p<0.01. (D-F) flow cytometric analysis of Ter119^+^, Gr1^+^, B220^+^ and CD3^+^ splenocytes in WT or JAK2V617F mice treated with vehicle or PF-13.

Splenomegaly is a characteristic phenotype of patients with MPN and JAK2V617F transgenic mice, and reduction in splenomegaly is a therapeutic endpoint in MPN clinical trials[40, 43]. We compared the effect of smoothened (SMO) antagonist (PF-13) (100 mg/kg) given by oral gavage on the spleen weights in JAK2 WT and JAK2V617F mice. As expected, untreated JAK2V617F mice exhibited pronounced splenomegaly (approximately three-fold) compared to JAK2 WT mice. SMOi resulted in a significant decrease in spleen size and weight in both male (average spleen weight: 343.5 milligrams (vehicle) versus 176.8 milligrams (PF-13)) and female (average spleen weight: 177.6 milligrams (vehicle) versus 110.3 milligrams (PF-13)) JAK2V617F mice (Fig. 1A–B). Flow cytometric analysis of splenocytes from mice expressing JAK2V617F showed significant increase in Ter119^+^ erythroid progenitors and myeloid (Gr1^+^) precursors compared with JAK2 WT mice (Fig. 1C–D). However, homozygous JAK2V617F caused a decrease in B220^+^ cells with no change in CD3^+^ cells (Fig. 1E–F). Upon treatment with PF-13, spleen cell composition almost returned to normal values with a large decrease in Ter119^+^ and Gr1^+^cells and an increase in the percentages of B220^+^ cells (Fig. 1C–F). These data suggest that SMOi reverses extramedullary hematopoiesis in JAK2V617F mice.

### Treatment with SMO antagonist decrease inflammatory cytokines in JAK2V617F mice

**Figure 2:**
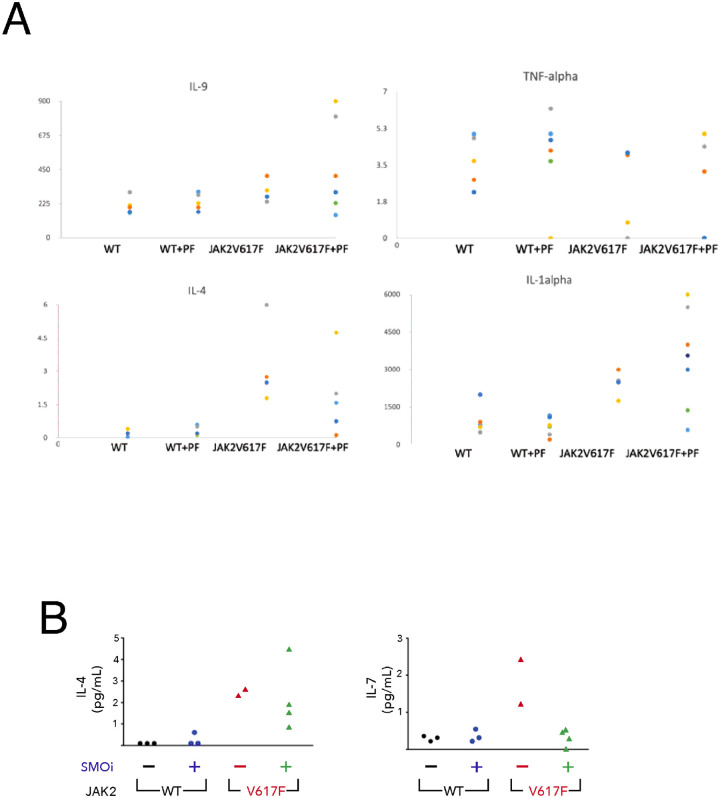
Treatment with SMO inhibitor PF-13 normalizes the cytokine levels of IL-4 and IL-7 in JAK2V617F mice. 8 week old WT or JAK2V617F mice were treated with vehicle or PF-13 (100 mg/kg) for five days. Serum was collected and cytokine levels were measured using the Mouse 32-Plex Cytokine Array.

The majority of MPN patients have the JAK2V617F mutation that constitutively stimulates the JAK-STAT pathway[44, 45]. Activated JAK-STAT pathway is known to enhance the production of inflammatory cytokines and growth factors. Overproduction of these cytokines in turn contributes to the expansion of the JAK2V617F mutated cells via cytokine receptor engagement[46, 47]. Indeed, elevated production of inflammatory cytokines including TNF-alpha, IL-2, IL-4, IL-6, CXCL9, and CCL2 was reported in JAK2V617F mutated cells[48, 49]. To identify cytokines that are altered in the JAK2V617F transgenic myelofibrosis mouse model, we measured the serum levels of 32 cytokines using the Mouse 32-Plex Cytokine Array in JAK2 WT and JAK2V617F mice. We identified a set of inflammatory cytokines, including IL-1alpha, IL-4, IL-6, IL-7, IL-9, IL-15 and TNF-alpha, which are elevated in the serum of JAK2V617F mice (Fig. 2A), similar to the alterations in circulating cytokines observed in patients with myelofibrosis[50]. Interestingly, short term PF-13 treatment (20 mg/kg, once daily for five days) normalized the levels of IL-4 and IL-7, which were elevated in serum samples of JAK2V617F mice (Fig. 2B).

### SMO antagonist PF-13 reduces JAK2V617F allelic burden

**Figure 3:**
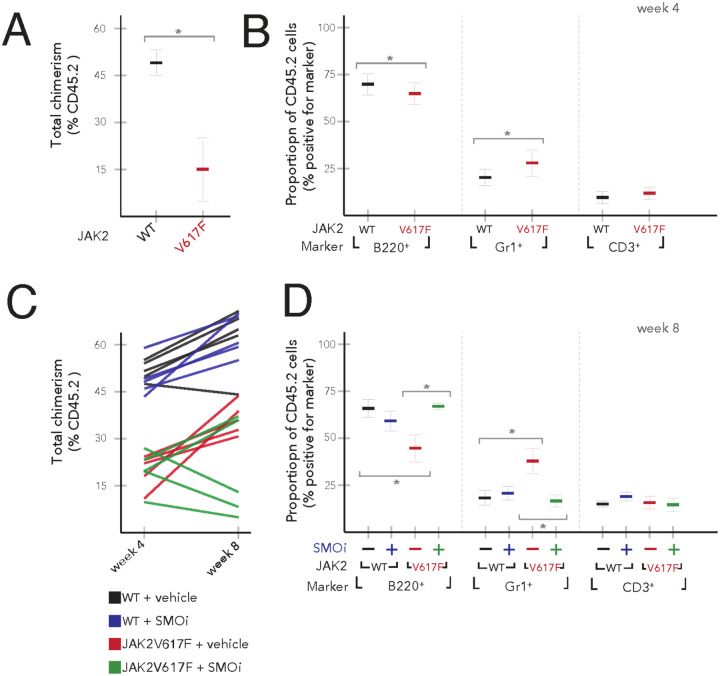
SMO inhibitor PF-13 reduces JAK2V617F allele burden. WT or JAK2V617F bone marrow was injected into lethally irradiated CD45.1 hosts. (A) Baseline chimerism at four weeks. (B) Frequency of B220^+^, Gr1^+^ and CD3^+^ cells at four weeks. (C) PF-13 treatment started at four weeks of initial bone marrow engraftment and CD45.2 donor chimerism checked at eight weeks. (D) Treatment with PF-13 normalized the frequency of Gr1^+^ (* p<0.05) and B220^+^ (*p<0.05) in JAK2V617F recipient mice.

Mutant JAK2 allele burden is a frequently used biomarker of disease burden. However, treatment with the JAK2 inhibitor Ruxolitinib results in only a minimal decrease of mutant JAK2 allele burden, suggesting that JAK2V617F cells are not dependent on JAK2 signaling for survival[14]. We investigated whether treatment with PF-13 decreases JAK2V617 mutant allelic burden *in vivo*. For these experiments, we made mixed chimeras by transplanting 1 × 10^6^ JAK2 WT or JAK2V617F BM cells (CD45.2) into lethally irradiated normal CD45.1 congenic hosts. Transplant efficiency was measured using anti-CD45.1 and anti-CD45.2 antibodies. We found that the mice receiving JAK2V617F BM cells (average percent CD45.2 cells: 21%) displayed reduced peripheral blood chimerism compared to those receiving JAK2 WT BM cells (average percent CD45.2 cells: 50%) (Fig. 3A). Flow cytometric analysis of the peripheral blood revealed that mice that received JAK2V617F BM cells showed a relative expansion of the myeloid compartment, with an increase in the Gr1^+^ population (approximately 2-fold) with a concomitant decrease in the B220^+^ B cell compartment as compared to mice receiving JAK2 WT BM cells (Fig. 3B). No difference was observed in the frequency of CD3^+^ T cells in mice receiving either JAK2 WT or JAK2V617F BM cells. Transplanted recipient mice were treated with PF-13 (100 mg/kg once daily) for one month and assessed for changes in peripheral blood chimerism. Four groups of mice were analyzed: (1) recipients of JAK2 WT BM cells treated with vehicle (WT + vehicle), (2) recipients of JAK2 WT BM cells treated with PF-13 (WT + SMOi), (3) recipients of JAK2V617F BM cells treated with vehicle (JAK2V617F + vehicle), and (4) recipients of JAK2V617F BM cells treated with PF-13 (JAK2V617F + SMOi). Treatment with PF-13 resulted in no change in JAK2 WT engraftment compared to controls. We found that PF-13 reduces the JAK2V617F allele burden in 3 of the 5 mice suggesting that PF-13 decreased the survival of JAK2V617F cells (Fig. 3C). Furthermore, treatment with PF-13 normalized the frequency of Gr1^+^ and B220^+^ in JAK2V617F recipient mice, reversing the myeloid skewing seen in JAK2V617F transplanted mice (Fig. 3D).

### Murine JAK2V617F splenocytes have increased levels of SHH

**Figure 4:**
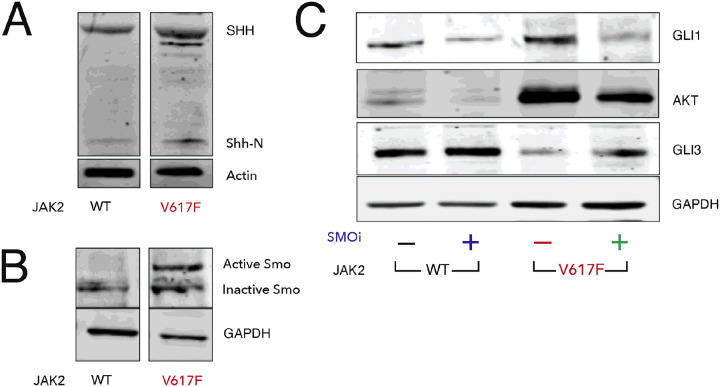
JAK2V617F increases SHH expression. Spleens from JAK2V617F transgenic and WT control mice were harvested and subjected to western blotting. (A) SHH precursor (45 kDa) and active Shh-N (19 kDa) are increased in JAK2V617F. (B) SMO is activated in JAK2V617F. (C) GLI activator (GLIA) and GLI repressor (GLIR) defines the output of Hh pathway. The increase in SHH ligand was consistent with the increase in the expression of Hh signaling targets such as GLI1 and AKT. Activation of Hh signaling was associated with a decrease in repressor GLI3 which increases with PF-13 treatment.

To better characterize the status of hedgehog signaling in JAK2V617F mice, we isolated protein from the spleens of 8-to-10 week old JAK2 WT and JAK2V617F mice, and measured levels of key hedgehog pathway members. Western blots for Sonic hedgehog (SHH), one of the activating ligands, showed increases of both precursor (SHH) and the secreted form (Shh-N) in JAK2V617F splenocytes (Fig. 4A). Pathway activation is mediated by Smo, and high levels of activation of Smo is associated with glycosylation that can be seen as a second band with reduced electrophoretic mobility in JAK2V617F tissue (Fig. 4B). Activation of hedgehog signaling was confirmed by the increase in the expression of Hh signaling targets such as GLI1 and AKT and decrease in the SMO regulated repressor GLI3. In previous work, we have shown that PF-13 decreases leukemia cell growth by increasing GLI3R levels and decreasing AKT levels[41]. When we treated JAK2 WT and JAK2V617F mice with PF-13, we observed increases in GLI3R with concomitant decreases in the Hh targets GLI1 and AKT (Fig. 4C).

### JAK2V617F is associated with increased pERK and NF-κB activation

**Figure 5:**
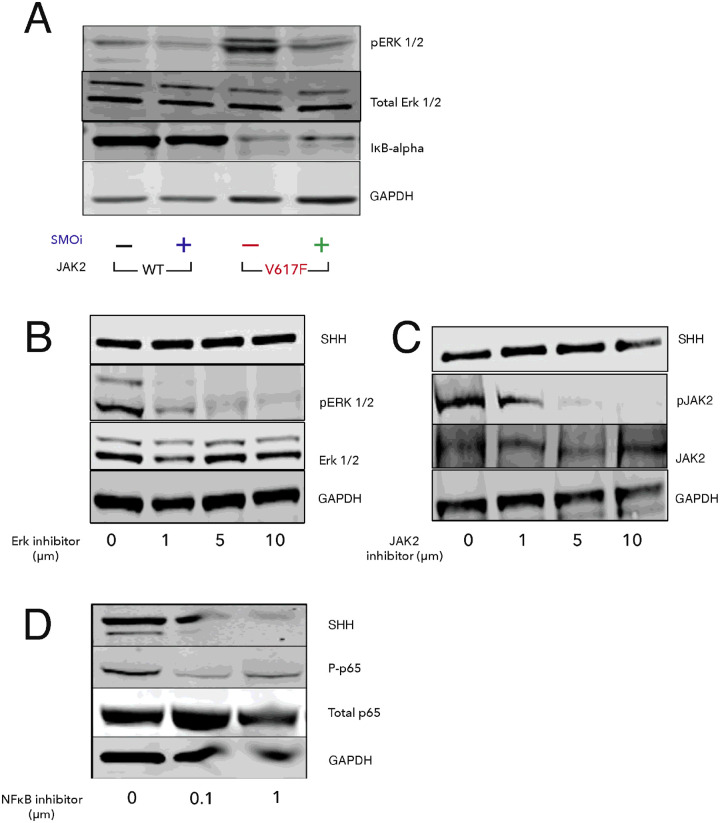
JAK2V617F activates NF-kB and MAPK signaling. MAPK and NF-kB is reversed by the Smoothened inhibitor, PF-13. (A) Spleens were harvested from JAK2V617F or WT mice after treatment with PF-913 (100 mg/kg) or vehicle for five days. Western blotting was done for pERK 1/2, Total ERK1/2, and IkB-alpha. (B-D) Splenocytes from JAK2V617F mice were cultured in the presence of ERK inhibitor, JAK inhibitor, or NF-kB inhibitor for 48 h, respectively. SHH levels were measured by western blotting. pERK 1/2, pJAK2, and phosphorylated p65 (P-p65) were also measured as controls for inhibition.

Previously, the ERK pathway was found to be activated in JAK2V617F MPN[51]. Recently, Fisher et al., [52] indicated that NF-κB signaling contributes to JAK2V617F mediated myeloproliferative disease in humans. To examine the activity of these pathways in our animal model, we assessed ERK and NF-κB activity by western blot. As expected, JAK2V617F mice showed increased pERK and NF-κB signaling, as evidenced by increased pERK ½, and decreased IκB-alpha, respectively (Fig. 5). Treatment with PF-13 normalized signaling of both these pathways suggesting that the therapeutic effect of PF-13 might in part be through modulation of ERK and NF-κB signaling pathways.

### SMO antagonist PF-13 blocks bone marrow fibrosis by reducing TGF-β signaling

**Figure 6:**
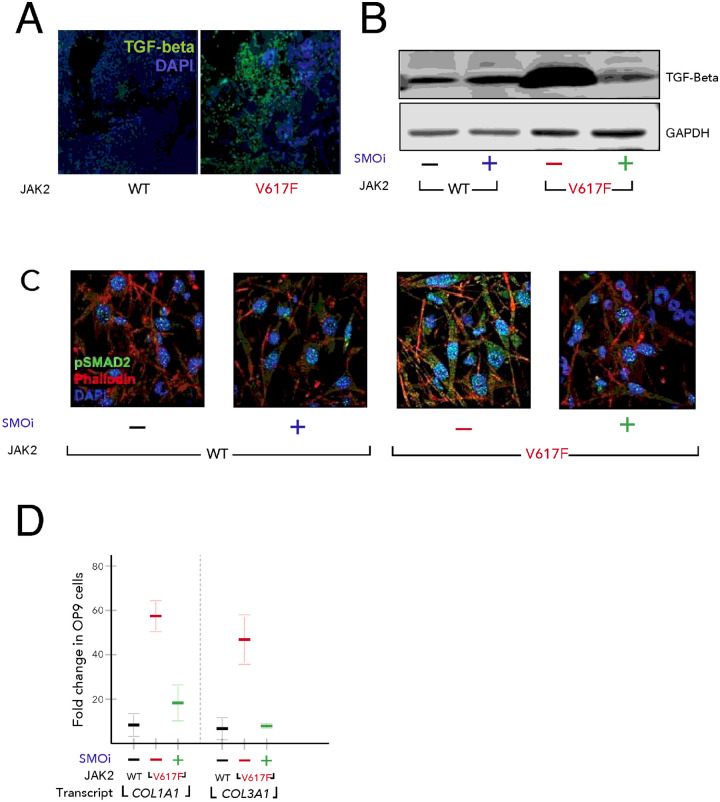
TGF-β induction is dependent on Hh signaling. (A) JAK2V617F bone marrow from patients show increased TGF-β (green) by IF. (B) TGF-β is induced in JAK2V617F mice and is normalized by PF-13. (C) JAK2V617F cells induce Hh dependent TGF-β/pSMAD2 signaling in the stroma. Splenocytes were co-cultured with OP9, and treated with either vehicle or PF913 for 24 h. Splenocytes were removed and stromal cells were stained for pSMAD2 (green), phallodin (red), and DAPI (blue) or (D) RNA harvested for expression of collagen genes - *COL1A1* and *COL3A1*.

Bone marrow fibrosis is a prominent feature of JAK2V617F associated myeloproliferative disease[53, 54]. Numerous studies have implicated the involvement of TGF-β in the pathogenesis of fibrosis[55, 56]. In our analysis, we found that bone marrow sections of JAK2V617F patients express high TGF-β levels (Fig. 6A). We also measured the levels of TGF-β in splenocytes of JAK2V617F and WT mice. As expected JAK2V617F splenocytes also expressed high levels of TGF-β, which decreased upon treatment with PF-13 (Fig. 6B). To determine if JAK2V617F could induce TGF-β signaling in the bone marrow stroma, we cultured the mouse stromal cell line OP9 on coverslips in six-well plates. After 24 h, OP9 cells were co-cultured with either JAK2 WT or JAK2V617F cells to assess the induction of pSMAD2 in the stroma. Immunofluorescence (IF) analysis revealed that JAK2V617F robustly induced pSMAD2, which was completely blocked by PF-13 (Fig. 6C), suggesting that JAK2V617F induces TGF-β signaling in stromal co-cultures in a paracrine manner. Next, we investigated whether increase in Hh ligand production in JAK2V617F mutated cells could induce TGF-β signaling pathway in the surrounding stroma and mediate bone marrow fibrosis. In this set of experiments we measured the levels of type 1 and type 3 collagen, which are transcriptional targets of TGF-β signaling[57] in OP9 cells cultured with either JAK2 WT or JAK2V617F cells in the presence or absence of PF-13. After co-culture for 24 h, JAK2V617F cells were removed, and RNA was isolated from the OP9 stromal cells. Quantitative real-time PCR (qPCR) revealed that JAK2V617F induced an approximately 50-fold increase in *COL1A1* and *COL3A1* gene expression in OP9 cells, which could be blocked by SMO inhibition (Fig. 6D).

**Figure 7:**
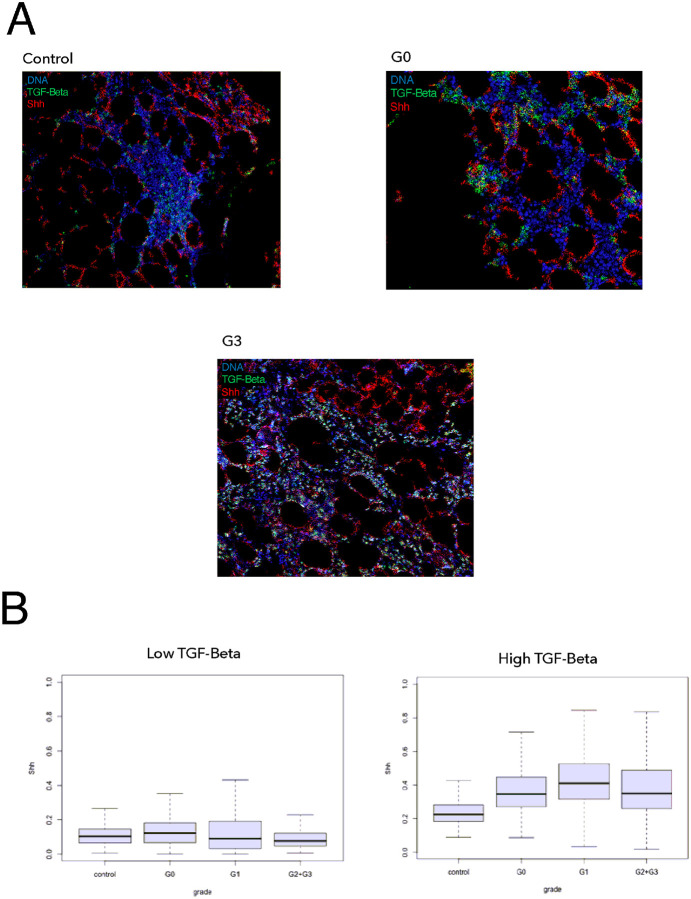
Higher grades of MF are associated with greater levels and increased co-localization of TGF-β and Shh in clinical samples (A) Example images generated by imaging mass cytometry on samples of normal and patient bone marrow samples with various (G0, G3) degrees of fibrosis. (B) Patch analysis of the images compared the amount of Shh present in areas high and low for TGF-β across different clinical grades of MF.

We were further able to integrate these data with that of clinical samples of patients with various grades of MF. We performed imaging mass cytometry on a cohort of 32 patients with various degrees of fibrosis[58]. Imaging mass cytometry (IMC) was chosen because it allows for precise quantification of protein unlike IHC, and does not have the problem of autofluorescence seen in IF on formalin-fixed paraffin-embedded (FFPE) bone marrow (Fig. 7A). To quantify the secreted proteins SHH and TGF-β, we used a novel patch based analysis rather than the cell segmentation approach typically used for IMC analysis[59]. This method allowed us to quantify spatial interactions of proteins at the pixel level rather than at the cellular level, which is better suited for extracellular proteins. Using this method, we observed increased localization of Shh in areas of relatively high TGF- β in patients with myelofibrosis when compared to control samples (Fig. 7B, p **< 0.001)**.

**Figure 8:**
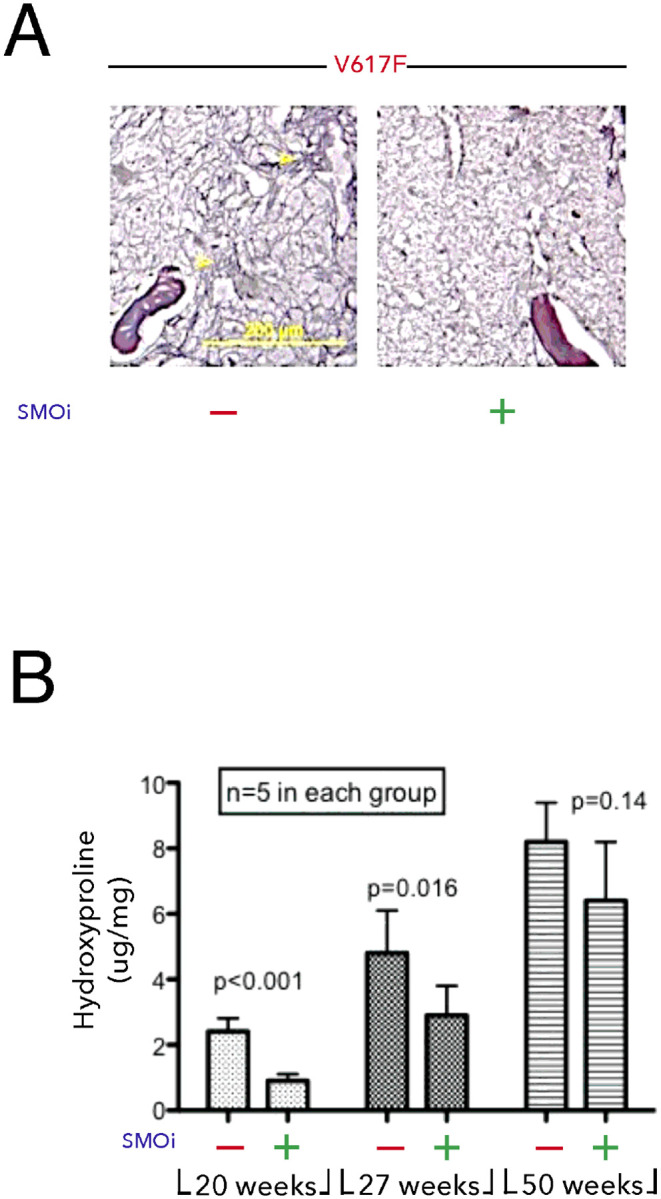
SMO inhibitor, PF-13, blocks fibrosis early. JAK2V617F transgenic mice were treated daily with PF-13 starting at 20, 27, and 50 weeks. After one month, bone marrow was stained for (A) reticulin and (B) quantification of hydroxyproline.

While JAK inhibitors demonstrate clinical benefit in patients with MPN, they have little effect on bone marrow fibrosis[60, 61]. Given our observation that blocking Hh signaling could modulate TGF-β expression and stromal collagen expression *in vitro,* we tested whether PF-13 could block bone marrow fibrosis *in vivo*. To assess for changes in bone marrow fibrosis, mice at 20, 27 and 50 weeks began daily treatment with PF-13 (100 mg/kg) for one month. Fibrosis was measured by reticulin staining and quantification of hydroxyproline[62]. The data demonstrated that treatment with PF-13 markedly decreased reticulin staining and was able to prevent fibrosis from developing in younger mice but was much less effective in reversing established fibrosis in older mice (Fig. 8A and B).

## DISCUSSION

In this study we establish that Hedgehog signaling is active in a mouse model of JAK2V617F MF and that modulation of Hh activity with the Smoothened inhibitor PF-13 had disease modifying effects. We found that GLI1 is upregulated and GLI3 is downregulated in JAK2V617F splenocytes, which also happen to produce high levels of Shh. It is unclear whether this GLI1 upregulation is due to canonical Hh pathway activation via Shh ligand or by aberrant activation elsewhere in the pathway. Studies from our lab and others point to the fact that Hh pathway output is dictated by the ratio of GLI activators (GLI1) and GLI repressors (GLI3)[21, 41]. Further, SMOi via PF-13 decreased Hh pathway activity by decreasing SHH and GLI1, and upregulating GLI3. Along these lines, we demonstrate that the JAK2V617F splenocytes showed active Hh signaling, which could be inhibited by treating JAK2V617F transgenic mice with PF-13. As expected JAK2V617F mice exhibited enlarged spleens, and SMOi led to significant decrease in spleen size for both male and female mice as well as a decrease in inflammatory cytokines.

In clinical trials, Ruxolitinib provides significant clinical benefit to JAK2V617F patients by reducing spleen size and lowering inflammatory cytokine levels, but has little effect on the malignant clone[16, 63]. In our model we demonstrated that treatment with PF-13 could reduce the JAK2V617F allelic burden.

JAK2 has the potential to activate multiple other signaling pathways such as MAPK, PI3K and NF-κB, either directly through downstream effectors, or indirectly through induction of target gene expression. In our transgenic JAK2V617F mouse model, we found activation of both ERK (in terms of phosphorylated ERK1/2) and NF-κB pathway (decreased IκB-alpha levels). Recently, Fisher et al., 2017[64] also observed NF-κB pathway hyperactivation in JAK2V617F associated myelofibrosis and secondary acute myeloid leukemia. The findings that SMOi decreased ERK and NF-κB pathway activity are novel and suggest that both ERK and NF-κB pathways act downstream of Hh signaling in JAK2V617F cells. It is noteworthy that Shh promoter region has putative NF-κB binding sites that specifically bind NF-κB complexes[52].

TGF-β is considered a master regulator of fibrosis of multiple tissue types[55, 56]. Elevated levels of TGF-β in JAK2V617F patients and splenocytes of JAK2V617F transgenic mouse model is a novel finding in this study. Intriguingly, SMOi decreased TGF-β levels suggesting that TGF-β could be a target of Hh signaling. SMOi proved equally effective in reducing BM fibrosis in JAK2V617F mice. Our co-culture experiments with JAK2V617F and OP9 cells demonstrated that Hh signaling pathway induced fibrosis by upregulating *COL1A1* and *COL3A1*.

There are several limitations to this study. The results are heavily dependent on a transgenic mouse model of JAK2V617F driven myelofibrosis; however, only half of patients with primary myelofibrosis have this characteristic mutation. The interaction between hedgehog signaling, MAPK and NF-kB, is intriguing and needs to be explored further. The current experiments do not clarify how these pathways interact in the context of mutant JAK2V617F signaling. Furthermore, the long-term *in vivo* studies on bone marrow fibrosis do not distinguish between the effect of PF-13 on mutant JAK2V617F blood cells or bone marrow stroma, which would need to be studied with stroma targeting genetic approaches to clarify the cell types being targeted by SMOi. Finally, it is noteworthy that clinical trials of SMOi as *single agents,* have not been successful in treating MPN, although reductions in bone marrow fibrosis were noted[27]. However these were all patients with advanced disease, and our mouse model suggest that earlier interventions might have greater benefit.

In conclusion, this study demonstrates a critical role of Hh pathway activation in the progression of MPNs. These data show that the SMO inhibitor, PF-13, inhibits Hh pathway activation by downregulating Shh and upregulating GLI3. Further, SMOi modulates TGF-β and MAPK signaling in MPN and ameliorates the severe phenotype (i.e., splenomegaly and BM fibrosis) in JAK2V617F mice and support the rational development of SMO and JAK inhibitor combinations in MPN.

## Data Availability

The datasets generated during and/or analyzed during the current study are available from the corresponding author on reasonable request.
